# Vaccines platforms and COVID-19: what you need to know

**DOI:** 10.1186/s40794-022-00176-4

**Published:** 2022-08-15

**Authors:** Isabel Acosta-Coley, Leonor Cervantes-Ceballos, Lesly Tejeda-Benítez, Lucellys Sierra-Márquez, María Cabarcas-Montalvo, María García-Espiñeira, Wilfrido Coronell-Rodríguez, Bárbara Arroyo-Salgado

**Affiliations:** 1 Biomedics, Toxicology and Environmental Research Group-BIOTOXAM, Cartagena, Colombia; 2 School of Medicine, Zaragocilla. Diag, 50 # 14-60. Laboratory Block No. 108 - No. 106, Cartagena de Indias, Colombia

**Keywords:** COVID-19, Vaccines platforms, Vaccine types, SARS-CoV-2, mRNA vaccines, Advantages and disadvantages, first–second- and third-generation vaccines

## Abstract

**Background:**

The novel SARS-CoV-2, responsible for the COVID-19 pandemic, is the third zoonotic coronavirus since the beginning of the 21 first century, and it has taken more than 6 million human lives because of the lack of immunity causing global economic losses. Consequently, developing a vaccine against the virus represents the fastest way to finish the threat and regain some "normality."

**Objective:**

Here, we provide information about the main features of the most important vaccine platforms, some of them already approved, to clear common doubts fostered by widespread misinformation and to reassure the public of the safety of the vaccination process and the different alternatives presented.

**Methods:**

Articles published in open access databases until January 2022 were identified using the search terms "SARS-CoV-2," "COVID-19," "Coronavirus," "COVID-19 Vaccines," "Pandemic," COVID-19, and LMICs or their combinations.

**Discussion:**

Traditional first-generation vaccine platforms, such as whole virus vaccines (live attenuated and inactivated virus vaccines), as well as second-generation vaccines, like protein-based vaccines (subunit and viral vector vaccines), and third-generation vaccines, such as nanoparticle and genetic vaccines (mRNA vaccines), are described.

**Conclusions:**

SARS-CoV-2 sequence information obtained in a record time provided the basis for the fast development of a COVID-19 vaccine. The adaptability characteristic of the new generation of vaccines is changing our capability to react to emerging threats to future pandemics. Nevertheless, the slow and unfair distribution of vaccines to low- and middle-income countries and the spread of misinformation are a menace to global health since the unvaccinated will increase the chances for resurgences and the surge of new variants that can escape the current vaccines.

## Introduction

**Fig. 1:**
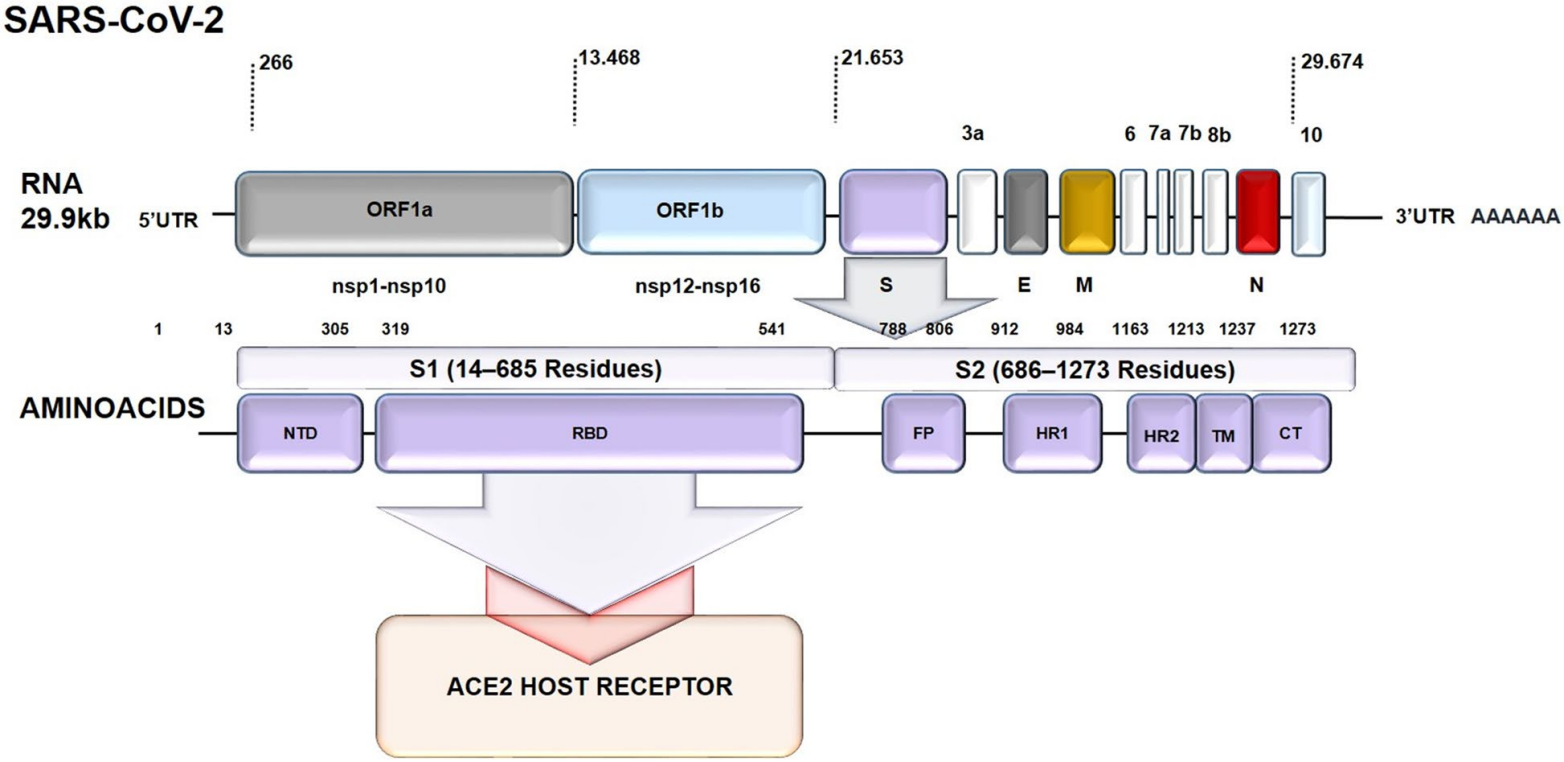
SARS-CoV-2 full RNA genomic schematic structure with approximately 29,700 nucleotides and S protein domains. The genome encodes two large genes ORF1a encodes (nsp1–nsp10), ORF1b encodes (nsp1–nsp16), for a total of 16 non-structural proteins (nsp1– nsp16). A replicase complex comprised by ORF1a and ORF1b at the 5′UTR region. Four structural genes encode the structural proteins, spike (S), envelope (E), membrane (M), and nucleocapsid (N) proteins, and accessory proteins included among the structural proteins, that are characteristic of SARS-CoV-2 in white. A poly (A) tail at the 3′UTR. NTD: N-Terminal Domain (14–305 residues). RBD: Receptor-Binding Domain (RBD, 319–541 residues). FP: Fusion Peptide (788–806 residues). HR1: Heptapeptide Repeat Sequence 1 (HR1) (912–984 residues). TM: Transmembrane Domain (1213–1237 residues). CT: Cytoplasm Domain (1237–1273)

The new Severe Acute Respiratory Syndrome Coronavirus 2 (SARS-CoV-2) is the agent responsible for the Coronavirus Disease 2019 (COVID-19), which was named that way by the World Health Organization (WHO) on February 11, 2020. The first outbreak was reported in December 2019 in Wuhan-China, and by January 30, it was declared a pandemic [1] due to the virus's high transmissibility and pathogenicity (Fig. 1) [2, 3]. As of 5:33 pm CEST, 1 June 2022, there have been 527,603,107 confirmed cases of COVID-19, including 6,290,452 deaths, and a total of 11,811,627,599 vaccine doses have been administered [4]. This event has been praised highly as the direst public health crisis of our times [5], and the third most highly pathogenic zoonotic beta coronavirus (β‐CoVs) of its kind, able to infect humans with pandemic potential since the beginning of the new millennium [6, 7].

COVID-19 is an emergent disease with many unknown aspects of its pathogenesis, and the main therapeutic approaches [8], consist of immunotherapies and antiviral drugs. Notwithstanding, the absence of a single specific antiviral therapy for CoV resides in its novelty since COVID-19 was unknown to humanity until recently. Hence, the treatments for the disease are mainly supportive. Nonetheless, a promising one is represented by plasma and antibodies obtained from convalescent patients [9, 10].

Because of its high mortality rate, an effective vaccine is fundamental and the best way to rapidly control the pandemic [11]. Thus, vaccines, in general, have become one of the most [12] cost-effective health interventions in recent history, saving approximately 2–3 million people each year contributing to supporting global health and the economy [13]. Even though the COVID-19 pandemic has entered its third consecutive year, the surge of highly adapted and transmissible Delta and Omicron variants alerts scientists about the consequences of vaccine escape mutations. Conditions that could deter vaccination efforts and have triggered global demands to intensify vaccination, including booster shots [14, 15].

Currently, COVID-19 vaccines are approved under a Biologics License Application (BLA) or authorized under an Emergency Use Authorization (USA) by the Food and Drug Administration (FDA) and recommended for primary vaccination by the Advisory Committee on Immunization Practices (ACIP) in the USA: the 2-dose mRNA-based Moderna and Pfizer-BioNTech/Comirnaty vaccines and the 1-dose adenovirus vector-based Janssen (Johnson & Johnson) COVID-19 vaccine [16].

The SARS-CoV-2 virus (Fig. 1) has a 30-kilobase positive single-stranded RNA organized into ten (10) specific genes that encode structural proteins and nonstructural proteins (NSPs). Structural proteins include spike (S), envelope (E), membrane (M), and nucleocapsid (N) proteins. The surface glycoprotein S interacts with the receptor for angiotensin-converting enzyme 2 (ACE2) in the host, allowing the virus to enter the cell. NSPs are generated as cleavage products of the viral open reading frame 1ab polyprotein (ORF1ab), facilitating viral replication and transcription. Among these polyproteins is RNA-dependent RNA polymerase (RdRp), known as Nsp12, a critical component that regulates the synthesis of viral RNA with the help of Nsp7 and Nsp8. It has other open reading frames (ORF3a, ORF6, ORF7a, ORF7b ORF8b, and ORF10) that encode five accessory proteins [17, 18].

During pre-pandemic times, a new vaccine design would take between 12 and 18 months, including clinical testing and regulatory consent [19], but creating a vaccine to prevent COVID-19 has become a global race between scientists and the virus [20]. The rapid progress in vaccine making has been facilitated in a record time by identifying the genome and structural information of SARS-CoV-2 [21, 22] and fundamental advances in epitope mapping and bioinformatics [23, 24], which have expanded the knowledge beyond traditional vaccine design. Thus, various vaccine platforms are developing, and since it is unclear which vaccine platforms will have the best performance, different strategies are in a trial [11, 25].

**Fig. 2:**
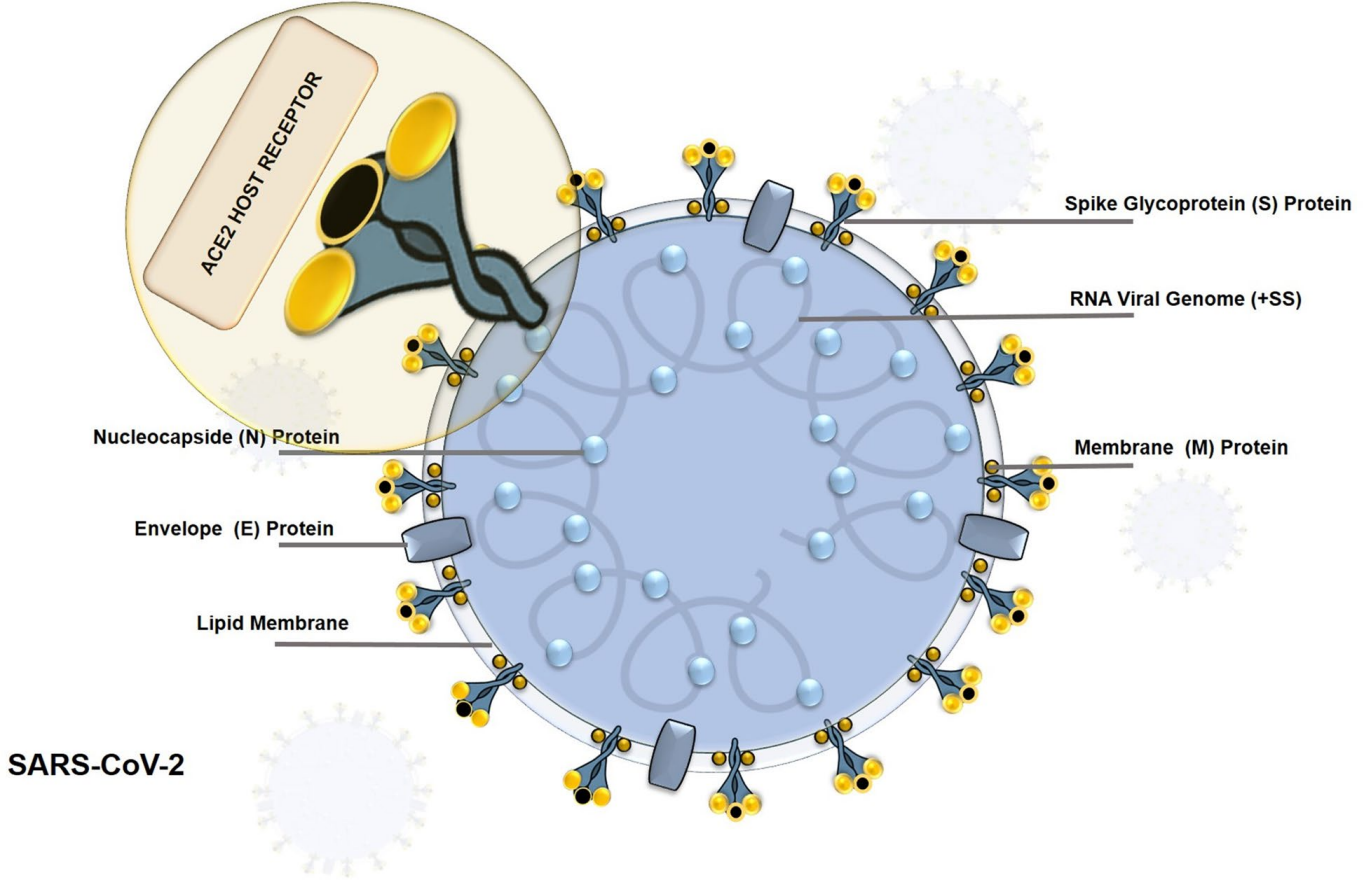
Structure of a spherical oily coated Beta-Coronavirus. A single-stranded positive-sense RNA (30 kb) surrounded by the (S), Envelope (E), and Membrane (M), and the enlargement of a Spike Protein (S), responsible for the entrance into the host cells via Angiotensin Converting Enzyme 2 (ACE2) human receptor

To reach herd immunity against SARS-CoV-2 (Fig. 2), global immunization coverage of ∼67% is estimated to be enough, supposing that the basic reproductive number (R0) (defined as the expected number of secondary cases produced by a single typical infection in a completely susceptible population) [26] of the virus is three [27]. Therefore, approximately ∼5.3 billion vaccine doses are required for a single shot or around 12–16 billion for vaccines requiring a booster shot, a solution that implies an enormous challenge. At this moment, the emergence of highly mutated variants endangers the immunity generated by the newly developed COVID-19 vaccines and increases the need for third or even more shots. This situation exacerbates the vaccine equity issue in low- and middle-income countries (LMICS) with a reduced purchase capacity and cannot compete with high-income vaccine hoarders [28–31].

As the pandemic continues, by January 11–2022, only 8.9% of people in low-income countries have received at least one dose [32], compared to more than 60% in high-income countries [33], which can secure advanced purchases from vaccine producers.

This review is intended for a broad scientific audience. It focuses on different vaccine platforms abridging their main advantages and disadvantages and summarizing key takeaway aspects of the COVID-19 vaccine formulations. It describes how each type works and boosts the body's immune response, leading to pathogen recognition and preventing lethal infection from SARS-CoV-2. Finally, it draws some conclusions to consider for the next pandemic.

## Methods

Open access databases, including PubMed/Elsevier and other relevant sources, including government health organizations, were searched for recent information updates. The articles published in English from database inclusion to December 10, 2022, using the search terms "SARS-CoV-2", "COVID-19", "CORONAVIRUS," "COVID VACCINES," "PANDEMIC," "LMICS" or its combinations. Before the COVID-19 disease outbreak in December 2019, there was no research on severe acute respiratory syndrome coronavirus 2 (SARS-CoV-2). The terms SARS-CoV and the Middle East respiratory syndrome coronavirus (MERS-CoV) were also consulted in this review with articles from 2002–2022. The language was restricted to English and Spanish.

## Vaccine types

Vaccines are considered a biological product developed to safely induce an immune response that will protect against infection and disease upon subsequent exposure to a pathogen. Usually, it consists of protein or polysaccharide antigens [34, 35]. They are often administered through needle injections, electroporation, a nasal spray, or by mouth [36]. The length and effectiveness of this immunity depend on the type, the virus (infectious), and the recipient.

One fundamental aspect is that vaccines target herd immunity [37], and immunization of a large piece of the population will protect immunocompromised, nonvaccinated, and immunologically naive individuals, people with underlying comorbidities, and those with severe allergies to some vaccine components. Therefore, vaccines decrease the number of unshielded people, reducing the transmission threshold [38–41].

**Fig. 3:**
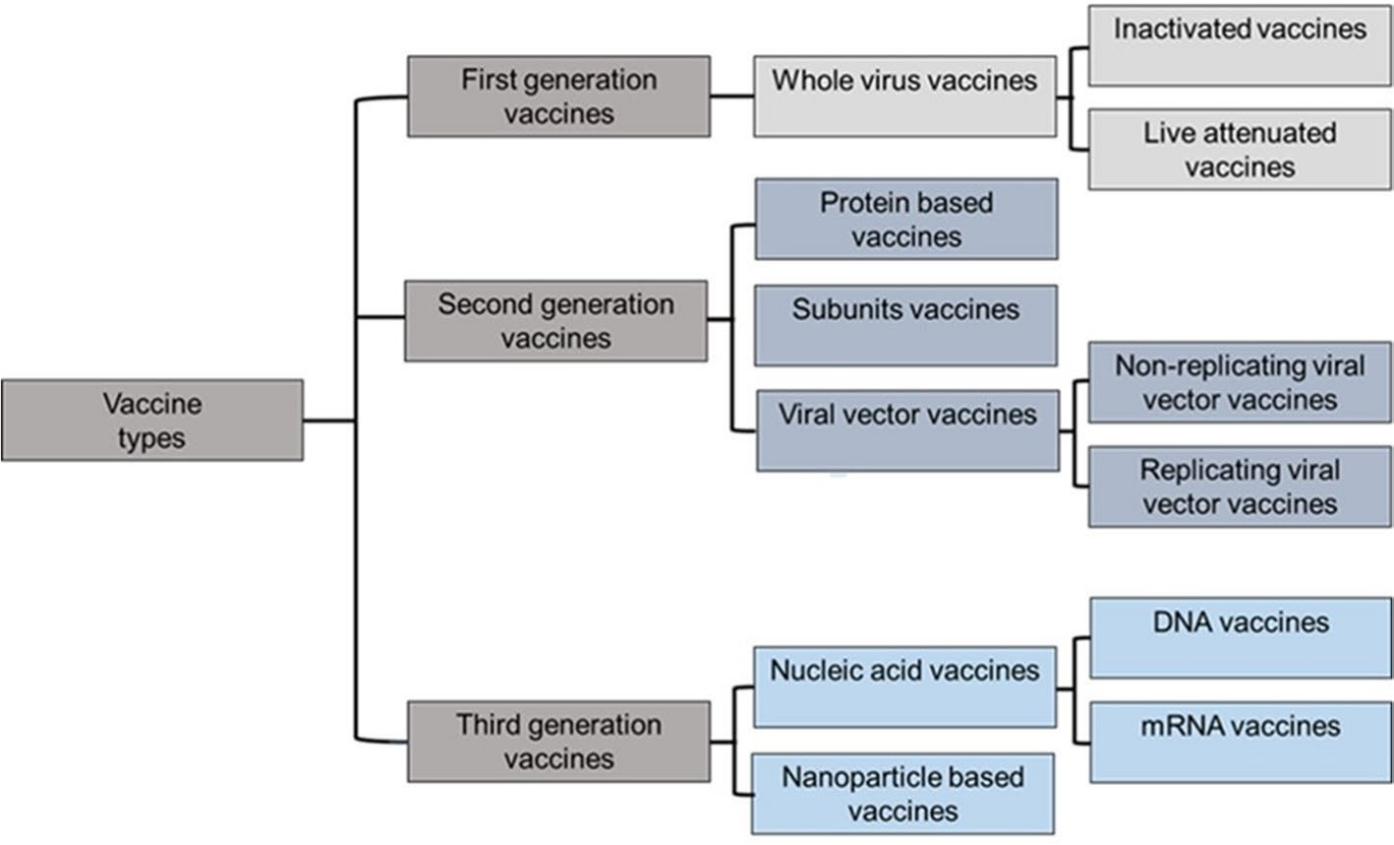
Main vaccines types and platforms for SARS-CoV-2

The current number of vaccines (Fig. 3) can be reduced into three categories: 1st-Generation Vaccines (FGVs), which employs the whole pathogenic microorganism like viruses and bacteria [42], and to avert the possibility of infection, the virus is attenuated or weakened by growing it in a different type. Additionally, microorganisms can be inactivated chemically (formaldehyde) or by heat; nevertheless, they can still stimulate all necessary signals for a strong immune response. As a substitute for the virus, 2nd-generation vaccines (SGVs) or recombinant vaccines use molecular biology to incorporate protein segments or subunits (Sub Unit Vaccines SUVs) [43], or whole viral proteins extracted from the pathogen. Lacking any genetic material inside, thus preventing replication, such as virus-like particles (VLPs). Alternatively, 3rd-Generation Vaccines (TGVs) (gene vaccines), based on genetic material DNA or RNA, are the front line for developing the COVID-19 vaccine. Instead of producing viral proteins or viruses on a massive scale under laboratory conditions, this platform directly injects plasmids into the recipient, coding for the target viral protein (the spike (S) protein) responsible for cell entrance in the case of SARS-CoV-2 (Fig. 2). Then, the host cells read the instructions and make the protein, producing the antigen in vivo, which elicits an immune response. One of its main characteristics is its remarkable adaptability to new pathogens [44–46].

Many of these approaches are not commonly the basis for vaccine development, but their success in other medical fields, such as cancer research, makes them very appealing. The immuno-informatics method is employed to identify the epitope for the SARS-CoV-2 vaccine candidates and recognize significant cytotoxic T-cell and B-cell epitopes in viral proteins [47, 48]. From another perspective, designed nanoparticles [NPs] play an essential role in targeting vaccine delivery to immune cells and improving vaccine efficacy, antigen uptake, and the induction of humoral and cellular responses [43]**.** NPs have played an essential role in activating antigen-presenting cells (APCs), especially dendritic cells (DCs), determining vaccine efficacy. Although there is some cytotoxic effect of the NPs [49, 50], the risk is low compared to the vaccine delivery benefits [51]. These approaches work very differently [52]**,** and they are now available to the scientific community for vaccine design [53] to conclude the final chapter in this pandemic [11].

In Latin America and the Caribbean, at least 53,352,000 reported COVID-19 infections and 1,572,000 deaths caused by the new coronavirus have been reported. Among the countries in the region, Argentina is in first place with the highest number of daily infections: 111,701 cases, while Brazil ranks first in the number of deaths/day with 215 [54]**.** Reports for Colombia show that there have been 5,624,520 infections and 131,437 deaths related to SARS-CoV-2 since the pandemic began [55]. According to Our World In Data (a project of the Global Change Data Lab), the proportion of people vaccinated against COVID-19 on 17 January 2022 is 76.96% of the population, of which 58.76% are fully vaccinated, 18.20% are partially vaccinated, and 8.8% have received booster doses [56]. The vaccines administered correspond to Pfizer-BioNTech, Oxford-AstraZeneca, Johnson & Johnson, Moderna, and Sinovac. However, there are no reports on the cumulative number of doses administered, broken down by the vaccine manufacturer for the country [55].

### First generation vaccines

#### Whole Virus Vaccines (WVVs)

Whole virus vaccines, either killed (IVV) or live-attenuated (LAV), have multiple antigenic components that potentially induce a wide variety of immunologic effectors in the host against the virus. [57] In WVVs**,** virions are irradiated or chemically inactivated and still contain abundant immunogenic components from the original virus, with no risk of reactivation under proper procedures [11]. Nevertheless, it is crucial to consider that the inactivation process could also lead to structural deformation of the immunogenic epitopes, disrupting the protection they afford [11].

This type of vaccine is considered highly efficient from the immunological perspective and exerts a wider cross-protection, potentially inducing mucosal, systemic, humoral, and cell-mediated immunity upon immunization [58–61]. The efficacy of these vaccines is higher in first-time host primary vaccination followed by a parenteral boost to improve the immune response [60].

Since WVVs from SARS-CoV and MERS-CoV trigger eosinophil-related lung pathology [62, 63], this vaccine type may be less attractive for developing a coronavirus vaccine. Although UV- and formaldehyde-inactivated SARS-CoV can elicit a neutralizing antibody response, the vaccine has been considered safe [64, 65]. Later, studies found that this type of vaccine, with or without adjuvants, did not confer complete protection in mice and led to eosinophilic pulmonary inflammatory response upon SARS-CoV exposure [62]. The same happens with gamma-irradiated adjuvanted MERS-CoV, which also ends up causing eosinophil-related lung pathology after viral exposure, even though it is capable of eliciting neutralizing antibodies [66]. These drawbacks are daunting. However, newly encouraging research combining different adjuvants and inactivation strategies showed that the whole inactivated virus is still an option for coronavirus vaccine development [11].

The best asset from WVVs is their natural immunogenicity and ability to stimulate toll-like receptors (TLRs) 3–7-8 and 9 of the innate immune system, which includes B cells and helper and killer T cells (CD4 and CD8 T cells, respectively) [67].

Some examples of clinically approved vaccines of this kind are hepatitis A, rabies, influenza, polio (Salk), plague, typhoid, cholera [11], rotavirus, yellow fever, and measles virus.

##### Inactivated Virus Vaccines (IVVs)

These vaccines also include the disease-causing virus, as a whole or in fragments, previously killed by heat or chemically inactivated [68]. Their genetic material has been destroyed; thus, the virus is no longer infectious and cannot cause disease. For this reason, they are labeled safer and more stable than live attenuated vaccines [69], and they can be administered to people with compromised immune systems.

Even though their genetic material has been destroyed, inactivated viruses usually contain many proteins to which the immune system can react. Inactivated vaccines only stimulate antibody-mediated responses, and this response is generally weaker and less long-lived because they cannot infect cells [70]. IVs are frequently administered alongside adjuvants (agents that stimulate the immune system), and booster doses may be required to overcome this problem [48, 71].

IVVs have been produced for a long time, and the procedure regarding their output is well established and relatively uncomplicated. Nevertheless, the process of virus inactivation must be adequate, without altering the viral proteins that could lead to a weak immune response or an enhanced disease after encountering the natural virus [52, 69, 72]. Consequently, their inactivation could render them less immunogenic. Thus, multiple booster doses might be needed to establish a maintained immunity memory, along with the presence of adjuvants [69, 73].

Sometimes the complete virus is used; other possibilities for the making include the fragmentation of the virus with detergents to break it or purifying the antigenic parts to create a subunit vaccine [74]. 

This vaccine requires special laboratory facilities [75] because the pathogen needs to be grown in continuous cell lines or tissues; hence, the virus should be purified and concentrated before chemical inactivation occurs. An example is the influenza virus typically grown in eggs to yield the inactivated influenza vaccine [72]**.** The use of adjuvants increases their immunogenicity, especially in older populations, due to immune senescence. Recent adjuvant improvements, such as MF-59 (Novartis), CpG 1018 (Dynavax) and AS03 (GSK), among others, are in use [48, 71]. Furthermore, vast numbers of viruses need to be handled, and the integrity of the immunogenic particles is maintained. Even though IVVs are more stable than LAVs, they still need a cold chain [68] and will probably require two or three doses to be inoculated [75].

Currently, several IVVs are being extensively used, including polio, rabies influenza, and hepatitis A [72], and several inactivated SARS-CoV-2 vaccines have been developed, including those by Sinovac Biotech, Sinopharm- the Wuhan and Beijing Institute for Biological Products [76].

##### Live Attenuated Vaccines (LAVs)

**Fig. 4:**
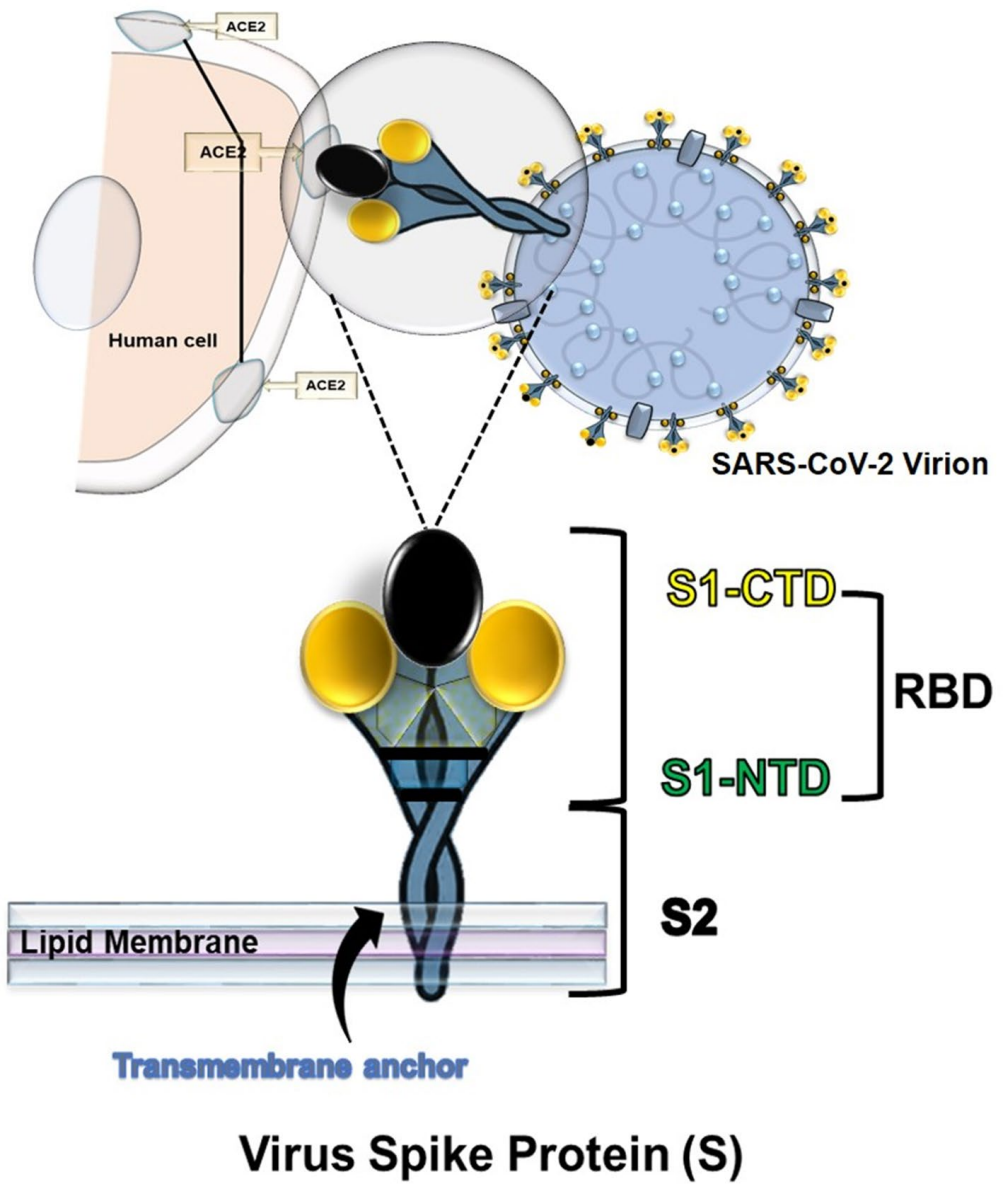
SARS-CoV-2 Virion and Spike protein subunits. Subunit 1 S1-CTD (Citoplasmic Domain). S1 NTD (N-Terminal domain). S2 Subunit 2. Receptor Binding Domain [ RBD]. Angiotensin-converting enzyme 2 (ACE2)

This vaccine is the most immunogenic of its kind; this does not require the use of adjuvants [77, 78] and has an extensive record of achievement in controlling various infectious diseases (Fig. 4) [79]. LAVs are cultured cells in a laboratory and then processed into a vaccine. Therefore, the virus cannot replicate easily in humans, giving the immune system enough time to learn how to react against this weaker form of the pathogen antigens, presenting them to the immune system as in natural infections providing long-lasting immunity [79].

Notwithstanding, it is still difficult to quickly develop and produce LAVs for SARS-CoV-2 because of the extended period and amount of data necessary to guarantee that critical factors responsible for its virulence are eliminated. The virus is also appropriately attenuated [80]. Maintaining consistent live vaccine stocks over time is also problematic [78, 81], mainly due to a loss of virus efficacy and reproductive potential. This vaccine requires cold chain distribution [82]. Another con of their development is the pressing need for BSL-3 (biosafety level-3) facilities for mass production since they need to propagate high volumes/titers of the pathogen and conduct extensive testing to ensure safety [83, 84]. Although LAVS are considered safe theoretically, an outbreak in poliomyelitis was associated with some formaldehyde-inactivated live vaccines produced by Cutter Laboratories in 1955 in the USA. It was considered a failed inactivation process that led to 40,000 new polio cases, including 200 cases of paralysis and ten deaths [69, 85].

A specific source of concern regarding SARS-CoV-2 comes from the fact that the virus can be present in feces from both infected and individuals vaccinated with the LAVS-SARS-CoV-2 vaccine, increasing the chances of infecting unvaccinated individuals [86, 87]; the LAVS-SARS-CoV-2 vaccine involves the risk of recombination with wild-type CoVs [41]**.**

The making of recombinant SARS-CoV-2 viruses from fragments of viral DNA has been made possible by newly developed synthetic genomics approaches [88, 89], which are employed to enhance the production of SARS-CoV-2 LAVs. Potential targets for these vaccines are the structural envelope protein (E), which is associated with aggravated inflammation in the lung [11, 90], and its removal can diminish CoV virulence [91, 92].

Another viable target is the nonstructural protein 16 (nsp16), which encodes ribose 2′-O-methyltransferase required for 5′ capping of viral RNA [93]**.** The methylation process facilitates coronavirus to avoid activating the type I interferon-dependent innate immune response by viral RNA; hence, nsp16 deletion weakens the virus [93]. The last target for LAVVs is nonstructural protein 14 [nsp14], which encodes exoribonuclease (ExoN) linked in RNA proofreading during replication [94]**.** Initial research on silent codon changes showed their influence in reducing events due to reversion, but this cannot be applied to all viruses [95]. Novel technologies such as genetic code expansion are developing more reproductive and, simultaneously, more stable LAVs [96]**.**

This design approach uses a single virus strain, which may not cross-protect against other strains. Particularly as the virus continues to disseminate worldwide, giving way to mutations as selection pressure rises [97], it will require/intend a single-dose immunity boost [79]**.** Three LAVs candidates are in the making by Mehment Ali Aydinlar University in Turkey, Codegenix/Serum Institute of India, Indian Immunologicals Ltd/Griffith University [98].

An associated risk is virus reversion or transfer to its pathogenic form. In immunodeficient patients, reactivation could befall. Therefore, biosafety issues of LAVVs require being evaluated with caution before proceeding to its clinical approval [79]**.** This vaccine is broadly contraindicated during pregnancy and in immunocompromised individuals [77, 78]. It could be temperature-sensitive, requiring proper storage [99]. It is also possible that recombination with similar circulating viruses in the population might occur, particularly for newly discovered diseases with unknown pathophysiology (Fig. 4).

Clinically approved examples of LAVs are mumps, measles, bacillus Calmette-Guerin (BCG), rotavirus, varicella, rubella, polio (sabin), yellow fever [11], smallpox and chickenpox.

## Second Generation Vaccines

### Protein-Based Vaccines (PBVs)

This type of vaccine relies on purified proteins (shells or fragments) obtained from a live or virus and directly or indirectly injected as a vaccine. The latter is through nanotransporters or encoded by NAVs [100, 101]. PBVs, instead of working with a fragment or a complete virus, produce antigenic peptides under laboratory conditions by recombinant methods [102]**.** Moreover, a cell culture technique employs yeast/insects to produce recombinant viral proteins. PBVs have a straightforward design, can be produced faster than other vaccines [103], and have an effective and safe long history behind them. PBVs consist of viral proteins and no genetic material (no genes).

This strategy has been developed for many SARS-CoV vaccines. This vaccine behaves similarly to IVVs, where proteins can induce a protective immune response from the host preventing disease; these vaccines might require multiple shots [104]. A significant advantage over WVVs is their safety, requiring the presence of adjuvants to enhance the immune response [105]**.**

Since PBVs sometimes do not induce potent CD8 T cell responses, they heavily depend on delivery systems and adjuvant mixtures [82, 106]**,** usually combining different types of cholesterol nanoparticles, such as Novavax SARS-CoV-2 vaccine, saponins, and lipids (phospholipids). Adjuvants containing aluminum in small amounts have been harmless and have been commonly used since the 1930s. Adjuvant mixtures enhance the immune response and are essential to vaccinate older individuals, from whom a lower immune response is expected. However, the presence of adjuvants can lead to more localized reactions, such as pain, chills, fever, redness, and soreness, than free adjuvanted vaccines [72].

Peptide-based vaccines have an attenuated immune response and fail to elicit cross-protection immunity against different viral strains and provide long-lasting immunity [98]. Nevertheless, when using whole proteins, their immunogenic potential increases. However, it will lose immunity whenever the protein changes its native conformation. Some known examples of PBVs are Shingles, Hepatitis B, and Pertussis Vaccines.

#### Subunit Vaccines (SUVs)

For this type of vaccine, tiny structural parts from the pathogen, such as specific viral fragments, are employed to elicit an immune response [82, 107]. These vaccines do not contain any other components of the pathogen, which makes pre-existing anti-vector immunity and reactivation of virulence impossible [108]. SUVs can be presented as protein nanoparticles or virus-like particles (VLPs) by recombinant expression [109]; thus, the immune system can recognize the particular pieces of a virus/bacteria acting as antigens. However, the presence of adjuvants is necessary to elicit a strong protective immune response [110]. This type is considered safer because there are no live components that can be quickly produced.

Almost all receptor binding domain (RBD) subunit vaccines in the development process against SARS-CoV-2, are based on the S protein or RBD of the S1 protein as antigens [1, 110]. Many vaccines in trials belong to this type, since an essential subunit of SARS-CoV-2 is the spike protein or S protein attached to the virus's exterior and plays a vital role in the infection process [52]. To interfere with binding to the ACE2 receptor in host lungs [111], SUVs against SARS elicit an immune response to the S protein. SUVs can make up viral protein cages, proteins attached to synthetic nanomaterials, and VLPs. It uses adjuvants or other delivery methods, in addition to other benefits derived from different nanocarrier platforms [112, 113]. A significant proportion of the SARS-CoV-2 candidate vaccines in development are based on subunits from the S protein and subunits from its RBD region [114] and adjuvants acting as immune enhancer molecules [104, 105].

Clinically approved vaccines include *Streptococcus pneumoniae*, *Haemophilus influenzae* type B, *Bordetella pertussis*, influenza, HPV, hepatitis B, herpes zoster, meningococcal, and pneumococcal disease.

### Viral Vector Vaccines (VVVs)

These vaccines contain genetically modified viruses that may or may not replicate but do not cause the disease. Recombinant viral vectors enable intracellular antigen expression and induce a robust cytotoxic T lymphocyte response, leading to the elimination of virus-infected cells. The advantages of viral vectors are mainly (a) high-efficiency gene transduction; (b) precise delivery of genes to target cells; and (c) induction of robust immune responses and increased cellular immunity [115]. However, viral vectors present some problems, such as pre-existing immunity against the vector caused by the production of neutralizing antibodies due to previous exposure to the virus [116].

VVVs use a modified virus, acting as "the vector" to submit orders to the cells, so antigens are presented to the target cells. VVVs can yield more powerful cellular immune responses than the recombinant protein vaccine [117]. The antigen inside the cells imitates a natural infection, eliciting antigen-specific solid humoral and cellular immune reactions by itself; thus, the presence of adjuvants is not required [118]. The recombinant viruses express specific heterologous antigens of interest in an unrelated modified virus. It employs mammalian viruses [119] that have been altered genetically and repurposed [110], poxviruses, and adenoviruses [120]. Human adenoviruses are the most used replicating viral vectors for SARS-CoV-2 vaccine development [119]. In any case, an inoffensive virus is employed, not SARS-CoV-2 [121]. 

To manufacture large quantities of VVVs, viral vectors, such as adenovirus/retroviruses, must be grown in cell cultures and then purified [72]. These are complicated stages because even though the upgrade of cell cultures and the elimination of pollutants are indispensable, they can also impact the success of viral vectors [118]. VVVs offer a long-term and high level of antigenic protein expression. Thus, they have great potential for prophylactic use since they induce and prime cytotoxic T cells (CTLs), ultimately eliminating virus-infected cells [53]. This type of vaccine also includes the vaccinia virus that was used to prevent smallpox [72] and is highly specific in delivering the genes (DNA form) to the target human cells, where viral proteins are made to induce the immune response [116].

Most VVV coronavirus vaccines select the spike S domain subunits or the spike protein S antigen; this selection was based on previous discoveries in SARS-CoV and MERS-CoV research [59, 61, 93, 122–124]. Hence, viral vector platforms tested for these two diseases have also been used in COVID-19 vaccines. Similar to modified vaccinia virus Ankara (MVA), Vesicular Stomatitis Virus (VSV), rabies, measles, parainfluenza, and human and nonhuman adenoviruses [125]. Some clinically approved vaccines based on VVVs are hepatitis B and human papillomavirus.

#### Nonreplicating viral vector vaccines (NRVVVs)

A category of viruses regularly adapted into a vector is the adenovirus, a group of approximately 50 common viruses responsible for causing the common cold, sore throat, diarrhea, fever, and pink eye. People are frequently exposed and can cause only mild illness. Therefore, the human immune system is very good at combating adenovirus infection [52]. The advantages of an adenoviral vector include scalability, extensive tissue tropism, and inherent adjuvant qualities [126, 127]. For the SARS-CoV-2 vaccine, weakened forms of adenovirus 5 and adenovirus 26 are being used.

VVVs for COVID-19 enter the cell and control the cell apparatus to produce innocuous pieces of the virus, specifically the S protein [121]. Human adenoviruses are the most used replicating viral vectors for SARS-CoV-2 vaccine development [119]. In any case, an inoffensive virus is employed, not SARS-CoV-2[121]. The antigen inside the cells imitates a natural infection, eliciting antigen-specific solid humoral and cellular immune reactions by itself; thus, the presence of adjuvants is not required [118].

Accordingly, weakened vectors cannot replicate/reproduce because essential genes are suppressed. This platform confirms the substantial immunogenic property of LAVs and the safety aspect of SUVs to induce cellular immunity in the host [110]. In addition to this advantage**,** viral vectors can include large insertions in their genome, facilitating antigen design [118].

Current SARS-CoV-2 viral vector vaccines use nonreplicating human or chimpanzee adenoviruses. As mentioned before, even though most of the designed VVVs target the S protein or RBD, one measles virus-based SARS-CoV-2 vaccine also targets the N protein [128].

Adenovirus vectors are well tolerated and highly immunogenic in most people [98]. This vaccine platform's efficacy will be diminished if the host has immunity against the vector due to previous exposure [126, 127], as occurred before in measles and adenovirus-based vaccines [126, 129]. This explains why animal adenoviruses can be employed as vectors instead of humans. Sometimes, chimpanzee adenoviruses are used, so pre-existing immune responses will not abridge vaccine efficacy [72]. The COVID-19 vaccine Janssen can prevent coronavirus in people 18 years and older. It is made up of another virus (adenovirus family) that has been altered to contain a gene responsible for making a protein found on SARS-CoV-2 [130]—administered as a single dose (0.5 ml) and a booster (0.5 ml) after two months.

Nevertheless, anti-vector immunity could be present after the vaccine's initial shot, even if a simian adenovirus vector is employed. Regardless of this concern, four of the five adenovirus-based SARS-CoV-2 vaccines in clinical trials are programmed to be given as a single dose. In contrast, other vaccines in trials require at least two doses [98].

**Fig. 5:**
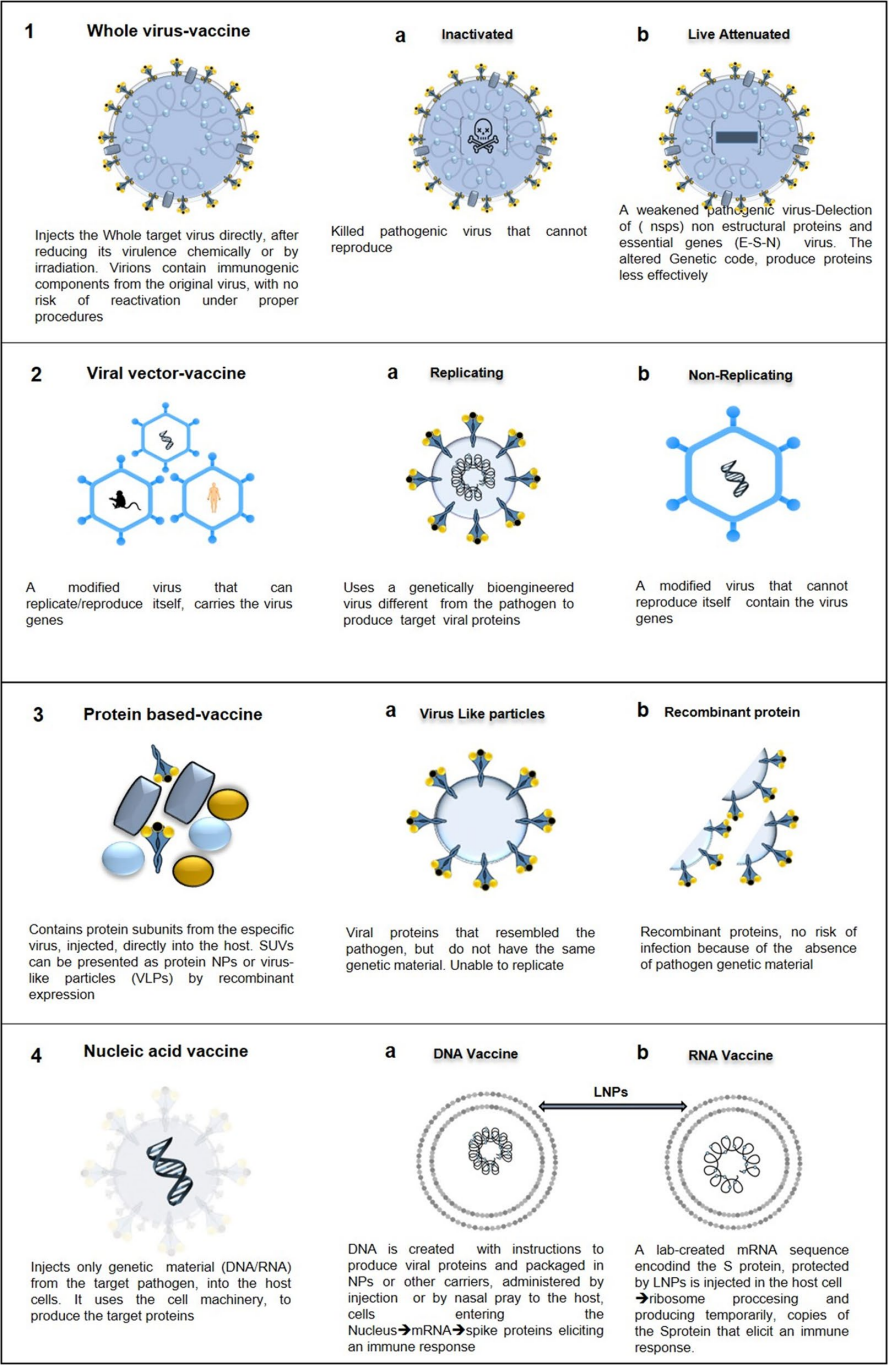
Vaccine front-runner candidates for SARS-CoV-2. Including Whole Virus, Viral Vector, Protein Based and Nucleic Vaccines

The AstraZeneca vaccine is an NRVV that encloses an inactivated cold virus with a code to produce S proteins (Fig. 5) that will be recognized as foreign, eliciting an immune response [52]. For the COVID-19 vaccine, researchers swap in a gene from SARS-CoV-2. Inside the host, the modified cold virus makes the SARS-CoV-2 protein, stimulating the immune response [131]. The DNA from the pathogen is not integrated into the host's genome; hence, it is transcribed into mRNA (messenger RNA), and then translated into proteins. Substantial risk is represented by the possibility that the recombinant viruses may integrate their genome into the human host, which requires previous biosafety assessment before clinical trials [126, 129]. These vaccines will likely need at least two doses.

#### Replicating Viral Vector Vaccines (RVVVs)

Associated disadvantages with RVVVs as a path to present genetic material to cells are the possibilities that the viral vector by itself can elicit an immune reaction in the body [132]. Additionally, previous exposure to the viral vector could make the vaccine useless. Unsuccessful vaccine testing on a patient implies that the same viral vector cannot be employed twice because of an immune reaction risk [132]. Genetic hazards can be surpassed using hybrid viral vectors [133] to improve transgenic expression levels. To overcome the issue of pre-existing immunity, the dose can rise, and the mode of administration [134] can be changed by exposing the individual to a nonviral DNAV [135].

RVVV platforms for the COVID-19 vaccine are horse pox, influenza, measles, and vesicular stomatitis viruses.

## Third generation vaccines

### Genetic Vaccines: DNA and Messenger RNA Vaccines (DNAVs-mRNAVs)

For the first time, in 1993, nucleic acid vaccines were tested, and in the same year, a liposome-entrapped messenger ribonucleic acid (mRNA vaccine) was used in mice to induce a virus-specific cytotoxic T lymphocyte response [136]. Genetic vaccines offer a cost-efficient and scalable approach to SARS-CoV-2 vaccine development [137]. Nucleic acid vaccines (NAVs) provide a whole new horizon, but until just recently, there was no licensed manufacturing platform. Nevertheless, recent advances have led to less innate immunogenicity, stability, and protein translation efficiency. These things and massive financial input have allowed the development of a new disruptive vaccine technology [138–140]. A foremost advantage of NAVs is that in addition to the antibody and CD4 + T cell responses, they elicit CD8 + cytotoxic T cell responses, which are essential for virus elimination [123, 141]. Immunization with NAVs upregulates the expression of crucial chemokines (CXCL9, CXCL10, and CXCL11) and cytokines (as type I interferons) that work by engaging dendritic cells and macrophages, such as immune cells that respond against expressed antigens, upgrading adaptive immune responses [142].

#### DNA Vaccines (DNAVs)

They are considered stable, safe to run [98], flexible, rapid, and attractive to combat emerging diseases such as SARS-CoV-2. Nucleic acids can be used to construct DNAVs containing genes that encode specific viral antigenic parts expressed by plasmid vectors and delivered into host cells. These vaccines can enter the nucleus and be translated into mRNA for protein synthesis [143, 144]. This type does not require handling the infectious pathogen and employs instead of the virus, a protein antigen, or a virus expressing the protein. Therefore, cells express the transgene, which provides a steady supply of specific proteins, similar to live virus responses [137].

DNAVs allow antigen-presenting cells to detect a tiny part of the virus without the cell previously absorbing and breaking down the live version of the virus or bacteria [52]. The former also presents the antigen to the immune system, ensuring that the recipient will not become ill when encountering the virus [52]. Vaccines such as this use 'normal' body cells instead of immune cells; this platform is a new technology, and to date, no vaccines for human use have been used [82, 98, 145].

DNAVs have better stability, are less fragile than mRNA vaccines and are exempt from the cold chain [146, 147]. Nevertheless, DNA vaccines are prone to be insufficiently immunogenic, and they cannot spread and amplify in vivo, requiring an indispensable prime-boost strategy to improve delivery and the presence of adjuvants to improve their strength [118]. Therefore, different mechanisms to enhance immunogenicity, e.g., nanotransporters such as VLPs, are being used, serving a double function and, in addition to behaving as enhancers, are meant to protect DNA. Other examples are immunostimulatory sequences and viral promoters [98]. Administration of DNAVs could be by intramuscular or intradermal injection, requiring efficient delivery methods to improve the DNA's intake, such as electroporation in vivo, jet injection without a needle, and the gene gun [118, 148]. 

DNAVs must cross two cellular membranes before reaching the nucleus and carry the risk of vector integration in the human genome, leading to dysregulated gene expression [98],possibly driving mutations and the development of cancer [110, 118, 149]. Although research has shown that the risk is shallow [98], WHO and the US FDA advise that studies on integration must be conducted as part of the safety program on DNA vaccines [150, 151].

The plethora of immunostimulatory mechanisms characteristic of DNAVs elicits humoral and cellular immune responses by activating CD8 + and CD4 + helper T cells and antibody production [98]. Upon entry into the cell, DNA vaccines are detected by various innate immune receptors, i.e., STING/TBK1/IRF3 pathways and the AIM2 inflammasome, among other factors involved in DNA vaccine mode action [118, 152, 153].

Immunization with S protein-encoded DNA vaccine elicited protective immunity against SARS-CoV infection in a mouse model by inducing T cell and neutralizing antibody responses [154].

Based on previous research on SARS-CoV, an exciting target for vaccine development is the main domain from the RBD of the spike protein, which can elicit neutralizing antibody and T-cellular immune responses against SARS-CoV infection [155, 156], a similar biological pattern that remains in SARS-CoV 2 [11].

#### Messenger RNA-Vaccines (mRNAVs)

Instead of a weakened or inactivated virus, RNAVs are a nucleotide-based novel vaccine platform [157] that incorporates viral antigen-encoding messenger RNAs that can be translated by cells to stimulate the immune system to manufacture target antigens [114, 121, 141, 144, 158–160]. Hence, the cell is fooled into manufacturing pieces or whole viral proteins, triggering a peculiarly effective and innate solid immune response characteristic of mRNA (Table 1) [159]. Extreme RNA replication occurs in the cytosol [161]; after triggering a response, the cell disintegrates and removes the mRNA [121].

**Table 1:**
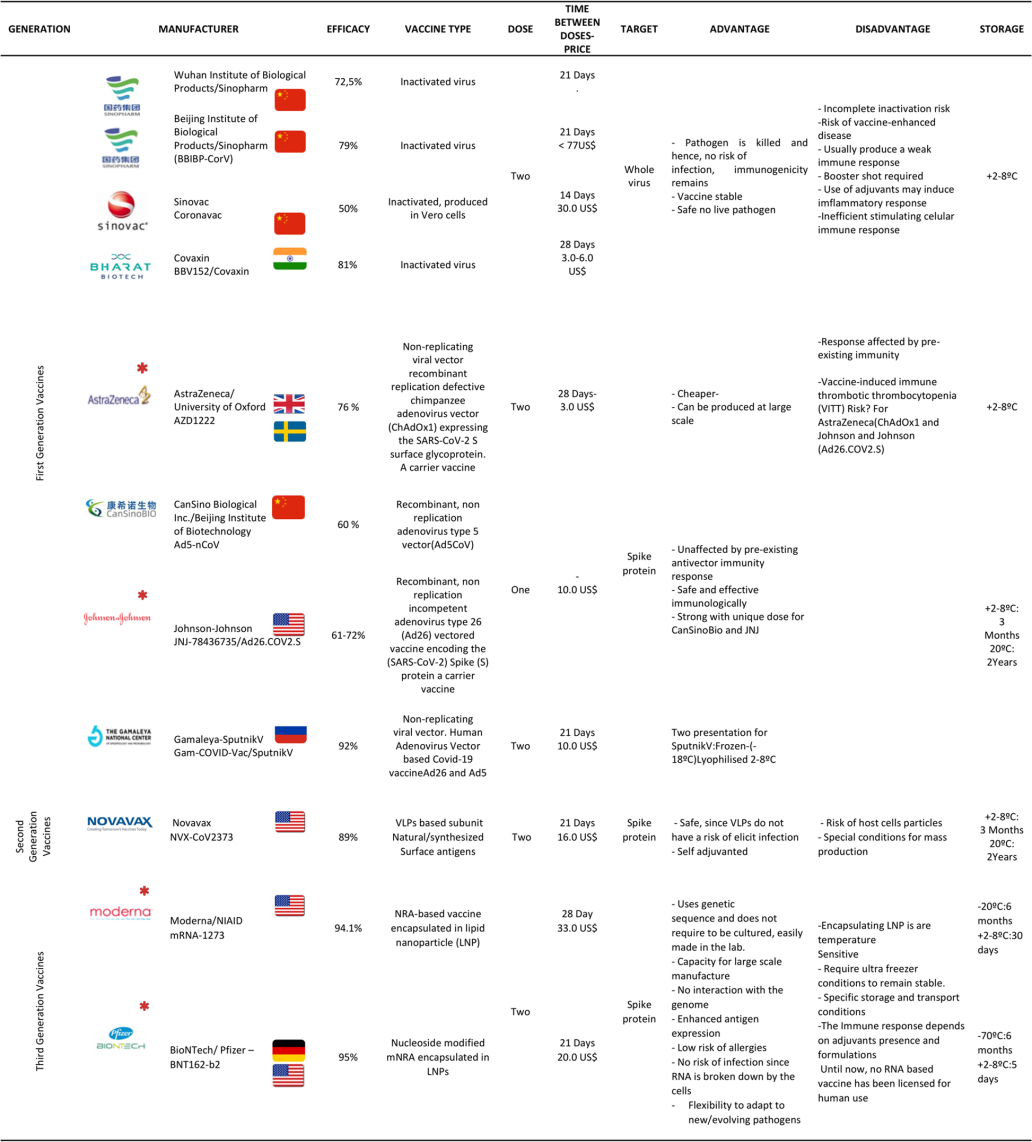
Main attributes of some approved vaccines including advantages and disadvantages. (*) Vaccines approved in the USA

The process of manufacturing RNAVs is chemical [144]. This vaccine type is considered an emerging, safer noninfectious, and nonintegrating platform[162]. Since it does not employ a pathogen or interact with the DNA in the nucleus [98, 121], this platform is a promising alternative to traditional vaccine design because of its high potency [98]. It offers adjustability and rapid vaccine manufacture in large quantities against newly discovered illnesses [162]. mRNA is exceptionally potent at inducing immune responses that quickly prevent the pathogen from spreading within the body, and mRNAVs can mimic the antigen structure and expression seen during natural infection [121, 159, 163].

These vaccines can be manufactured at a low cost, which is essential during this pandemic [60, 98] and is considered a new technology never used before in humans. Until recently, it has only been used to treat cancer since mRNA induces the immune system to fight carcinogenic cells [121, 145, 160].

A significant problem encountered by mRNA vaccines includes the need for packaging, which is why RNAVs are associated with additives, such as protamine or lipid- and polymer-based nanoparticles (liposomes), which raise their potency [158], for transport and protection [98].

Nanotechnology enhances the delivery of RNAVs in multiple ways, such as traditional intravenous injection, intradermal or intramuscular, using nanoparticles coated with lipids [60, 118]. Lipid nanoparticles (LNPs) are adopted to protect mRNA since mRNA is a sizeable hydrophilic molecule that cannot penetrate the cell alone; thus, vaccines use nanoparticles to ease their entrance into the cells where they are translated into proteins [160]. Therefore, crossing the lipid membrane barrier is the first step for exogenous mRNA to reach the cytoplasm before the translation of functional proteins occurs [164]**.** Different nanotechnology platforms, such as cationic nanoemulsions, dendrimers, polysaccharide particles, or liposomes, have been used to improve mRNA-based vaccine stability and transport [141, 165]. In 2018, the USFDA approved the first lipid nanocarriers for RNA to deliver a type of RNA called siRNA in charge of muting specific genes, causing disease. mRNA-1273 is a newly designed LNP-encapsulated mRNAV that encodes the spike protein (S protein) of SARS-CoV-2, created by Moderna® in the United States (US) and the (VRC) Vaccine Research Center of the (NIAID) National Institute of Allergy and Infectious Diseases [166, 167].

The hypothetical benefits of mRNA seem to outweigh their cons, as delivery and stability are related to RNA degradation and immunogenicity [168].  mRNAVs are less stable, requiring an uninterrupted extra cold-chain (ultrafreezer) process for transport and storage for wide-scale delivery [25, 145, 147] because, at high temperatures, mRNA can denaturalize since it is precarious under physiological conditions. The main reason for this is the presence of extracellular ribonucleases that catalytically hydrolyze RNA; therefore, the use of "unprotected" mRNA is not advised. Additionally, the RNA-negative charge and its hydrophilic nature make it difficult to embody the cells [141] effectively.

MRNAVs encode spike protein segments, which are much easier to reproduce under laboratory conditions than the whole spike protein.

Another issue is that exogenous RNA can activate an interferon-mediated antiviral immune response, which can interfere with the translation step and lead to mRNA damage, which removes the efficacy of RNAVs [162]; in addition, interferon signaling is linked to possible autoimmunity [141]. Until now, there has been no evidence of autoimmune illnesses connected to RNAVs, but close monitoring of the vaccination process is highly recommended due to the possibility of potential adverse results.

The injection of nanoencapsulated mRNA into the cells converts the host body into a factory [160]. For the COVID-19 vaccine to be effective, at least two doses will be needed. However, the multidose vaccine strategy involves the risks that many individuals who will agree to receive the first dose of the vaccine will not receive the second. This issue can be omitted by using novel delivery systems such as dissolvable microneedles that can administer minimal amounts, avoid possible side effects, and limit exposure to other vaccine components, which can also elicit an allergy reaction, such as LNPs [169].

The first approved mRNA German BioNTech/Pfizer's vaccine needs storage at − 70 degrees Celsius (− 94 degrees F), while the mRNA Moderna vaccine needs storage at − 20º (− 4 F), but lyophilization and the use of stabilizers allow the storage of some mRNA vaccines in a refrigerator instead of a freezer [160]. The former vaccine is approved for groups between 12–17 years old, with 2 doses of 30 μg (0.3 ml), 21 days apart, as well as for 18 years and older, which in contrast can receive a booster shot of 30 μg (0.3 ml) after 6 months [16].

The American pharmaceutical Moderna received approval from the U.S. FDA for its emergency use. Been the second vaccine for COVID-19, distributed in the U.S. for use in individuals older than 18 years [170]. With 2 doses of 100 μg (0.5 ml), 28 days apart, and a booster shot 50 μg (0.25 ml) after 6 months [16]. Moderately to severely immunocompromised individuals aged ≥ 12 years (Pfizer-BioNTech vaccine recipients) or ≥ 18 years (Moderna vaccine recipients) should receive an extra homologous dose of mRNA COVID-19 vaccine (same initial vaccine product administered previously) ≥ 28 days after the second dose. Any authorized COVID-19 vaccines (Pfizer-BioNTech, Moderna, or Janssen) may be used for the booster dose, regardless of the vaccine received for primary vaccination [16, 171]. For ages 12–17, only Pfizer BioNTech can be used as a booster dose, and the use of an mRNA vaccine for a booster dose is preferred over the Janssen vaccine [171].

Moderna's mRNA-1273 vaccine encodes a stabilized prefusion spike trimer [172], whose nucleotides were altered to avoid startup genes linked to interferon when entering the cell [172] and incrementally the half-life and translation process. These two pharmaceuticals are the leading designers for SARS-CoV-2 mRNAV. Both mRNA vaccines contain a protective envelope of lipids with an instruction that tells the cells to make spike proteins marked as alien, starting an immune response [52].

## Nanoparticle Vaccines (NPVs)

NPVs are considered safe, highly immunogenic, and stable[173], an essential advantage over less immunogenic and labile vaccines such as genetic vaccines (mRNAs-DNAVs) [82, 173]. NA and proteins and other specific antigens can be transported on the surface or inside NPs to elicit innate and adaptative immunity [82].

Among the materials employed to make nanoparticle vaccines are lipids, proteins, metals such as gold, VLPs, e.g., gold, and polymers that also behave as adjuvants [174]. The nanoparticle surface can be altered to target particular cells or enhance immunogenicity and packaging with TLR ligands and other immune modulators.

One of the most popular viral vaccine development approaches is engineering VLPs consisting of a self-assembled viral membrane in a monomeric complex that displays the viral epitopes but lacks multiple key viral components, ensuring no replication capacity [100].

## Conclusions

Humanity faces another coronavirus disease from zoonotic origins that will not be the last, since habitat destruction will continue to increase, along with an unsustainable growing population. Nevertheless, advances in different scientific fields, such as molecular medicine, bio and nanotechnology, and other biological areas have ensured the development of next-generation vaccines, which has given us a sense of confidence to handle upcoming diseases and novel pathogenic microorganisms, especially now, with the emergence of new and more infectious variants with challenging mutations.

Sequence information from SARS CoV-2 obtained in a record time provided the basis for developing new vaccine platforms and detecting variants of concern. This speedy achievement allows us to react faster to biological threats because of their adaptability, enabling us to respond faster to future pandemics.

Vaccines based on mRNA (by BioNTech and Pfizer) can be quickly manufactured under standard laboratory conditions, saving time, and lowering COVID-19 transmission. Nonetheless, due to their instability, the currently approved vaccines require storage in ultra-freezers or freezers and have short shelf lives once they are removed from storage. This asset means “no problem” for developed countries but is posing logistical nightmares for storage, distribution, and administration in LMICS since thigh coordination measures are required between all stakeholders to ensure an efficient distribution [175]. Consequently, the world will need more than one kind of vaccine, so different formulations should be designed to improve thermostability, among other things.

The CEPI (Coalition for Epidemic Preparedness Innovations), responsible for coordinating the global COVID-19 vaccine development effort, and the WHO and Gavi (Vaccine Alliance) have developed the COVAX facility to ensure equitable global access to COVID-19 vaccines. Nevertheless, despite its efforts, the disparity in access to vaccines is remarkable since high-income nations have secured more than half of the total doses of COVID-19 vaccine doses. In contrast, LMICS can cover just one-third of its inhabitants. Aside from this, some LMICs are not fully prepared to handle large-scale efforts to vaccinate their citizens and have the most significant discrepancy among the dose proportion purchased and population share compared to high-income nations. A fair vaccine distribution among all countries regardless of political or economic interest must prevent lethal outcomes and ensure control over the pandemic. Therefore, the responsibility to make this feasible, lies in the hands of the wealthiest nations; thus, the COVID-19 threat in overexploited countries does not become a threat to global efforts to reach population immunity.

Strategies to closely monitor possible undesirable side effects from vaccines must be implemented to reassure and grow confidence in the vaccination process.

An example of these side effects is the immunological response to vaccination, which could lead to antibody-dependent disease enhancement (ADE), possibly inducing a cytokine storm when the host becomes infected in the future after taking the vaccine [176] or vaccine-induced thrombotic thrombocytopenia (VITT) reported for AstraZeneca (ChAdOx1) and Johnson and Johnson (Ad26.COV2. S) [177].

Here, we provided information regarding different vaccine platforms to lessen vaccine hesitancy. The need to develop a vaccine in a record time might have given the wrong sense of overlooked risks and loose ends regarding its safety and effectiveness. Last, it is crucial to consider that refusing vaccination for some population segments will increase the probability of virus transmissibility and resurgences. The failure to supply COVID-19 vaccines to LMICs would encourage the surge of SARS-CoV-2 variants among them, with dreadful consequences.

## Data Availability

This does not apply to this article as no datasets were generated or analyzed during the current study.

## References

[1] World Health Organization. COVID-19 vaccine development, Coronavirus Update 37. World Health Organization. 2020 p. 28. Available from: https://www.who.int/docs/default-source/coronaviruse/risk-comms-updates/update37-vaccine-development-esc0a81735cd754b32b69ed4147cbbddec.pdf?sfvrsn=2581e994_33 cited 10 May 2021.

[2] Munster VJ, Koopmans M, van Doremalen N, van Riel D, de Wit E (2020). A novel coronavirus emerging in china — key questions for impact assessment. N Engl J Med.

[3] Zhou P, Yang X Lou, Wang XG, Hu B, Zhang L, Zhang W (2020). A pneumonia outbreak associated with a new coronavirus of probable bat origin. Nature..

[4] World Health Organization. WHO Coronavirus (COVID-19) Dashboard. Available from: https://covid19.who.int/ cited 2021 Apr 13.

[5] Chang L, Yan Y, Wang L (2020). Coronavirus Disease 2019: Coronaviruses and Blood Safety. Transfus Med Rev.

[6] de Haan CAM, Rottier PJM (2005). Molecular Interactions in the Assembly of Coronaviruses. Adv Virus Res.

[7] Wang C, Horby PW, Hayden FG, Gao GF (2020). A novel coronavirus outbreak of global health concern. Lancet.

[8] Dong L, Hu S, Gao J (2020). Discovering drugs to treat coronavirus disease 2019 (COVID-19). Drug Discov Ther.

[9] Ahn JY, Sohn Y, Lee SH, Cho Y, Hyun JH, Baek YJ (2020). Use of convalescent plasma therapy in two covid-19 patients with acute respiratory distress syndrome in Korea. J Korean Med Sci.

[10] Ye M, Fu D, Ren Y, Wang F, Wang D, Zhang F (2020). Treatment with convalescent plasma for COVID-19 patients in Wuhan. China J Med Virol.

[11] Der LY, Chi WY, Su JH, Ferrall L, Hung CF, Wu TC (2020). Coronavirus vaccine development: from SARS and MERS to COVID-19. J Biomed Sci.

[12] Greenwood B (2014). The contribution of vaccination to global health: Past, present, and future. Philos Trans R Soc B Biol Sci.

[13] World Health Organization. Coronavirus disease 2019 (COVID-19) situation report-73. 2019. Available from: https://apps.who.int/iris/handle/10665/331686?locale-attribute=es& 35767666

[14] Volz E, Mishra S, Chand M, Barrett JC, Johnson R, Geidelberg L (2021). Assessing transmissibility of SARS-CoV-2 lineage B.1.1.7 in England. Nature.

[15] Dolgin E. Omicron is supercharging the COVID vaccine booster debate. Nature. 2021; Available from: https://www.nature.com/articles/d41586-021-03592-2 10.1038/d41586-021-03592-2 34862505

[16] Mbaeyi S, Oliver SE, Collins JP, Godfrey M, Goswami ND, Hadler SC (2021). The Advisory Committee on Immunization Practices’ Interim Recommendations for Additional Primary and Booster Doses of COVID-19 Vaccines — United States, 2021. MMWR Morb Mortal Wkly Rep.

[17] Toyoshima Y, Nemoto K, Matsumoto S, Nakamura Y, Kiyotani K (2020). SARS-CoV-2 genomic variations associated with mortality rate of COVID-19. J Hum Genet.

[18] Rastogi M, Pandey N, Shukla A, Singh SK (2020). SARS coronavirus 2: from genome to infectome. Respir Res.

[19] Verch T, Trausch JJ, Shank-Retzlaff M (2018). Principles of vaccine potency assays. Bioanalysis.

[20] Lurie N, Saville M, Hatchett R, Halton J (2020). Developing Covid-19 Vaccines at Pandemic Speed. N Engl J Med..

[21] Wrapp D, Wang N, Corbett KS, Goldsmith JA, Hsieh CL, Abiona O (2020). Cryo-EM structure of the 2019-nCoV spike in the prefusion conformation. Science (80).

[22] Yan R, Zhang Y, Li Y, Xia L, Guo Y, Zhou Q (2020). Structural basis for the recognition of SARS-CoV-2 by full-length human ACE2. Science.

[23] Lucchese G (2020). Epitopes for a 2019-nCoV vaccine. Cell Mol Immunol.

[24] Ahmed SF, Quadeer AA, McKay MR (2020). Preliminary Identification of Potential Vaccine Targets for the COVID-19 Coronavirus (SARS-CoV-2) Based on SARS-CoV Immunological Studies. Viruses.

[25] Koirala A, Joo YJ, Khatami A, Chiu C, Britton PN (2020). Vaccines for COVID-19: The current state of play. Paediatr Respir Rev.

[26] Jones Holland J. Notes On R0. Available from: https://web.stanford.edu/~jhj1/teachingdocs/Jones-on-R0.pdf cited 17 Aug 2020

[27] Kwok KO, Lai F, Wei WI, Wong SYS, Tang JWT (2020). Herd immunity – estimating the level required to halt the COVID-19 epidemics in affected countries. J Infect..

[28] Torres I, Lopez-Cevallos D, Artaza O, Profeta B, Kang J, Machado CV (2021). Vaccine scarcity in LMICs is a failure of global solidarity and multilateral instruments. Lancet.

[29] Usher AD (2020). COVID-19 vaccines for all?. Lancet.

[30] Staff R. Amnesty: rich countries have bought too many COVID-19 vaccines. Reuters. 2020; Available from: https://www.reuters.com/article/health-coronavirus-vaccines-idINKBN28J1BY

[31] Berkley S. COVAX explained. Gavi, the Vaccine Alliance. 2020; Available from: https://www.gavi.org/vaccineswork/covax-explained

[32] Coronavirus (COVID-19) Vaccinations. Available from: https://ourworldindata.org/covid-vaccinations

[33] Maxmen A (2021). The fight to manufacture COVID vaccines in lower-income countries. Nature..

[34] The U.S. Food and Drug Administration. Vaccine Development – 101. Available from: https://www.fda.gov/vaccines-blood-biologics/development-approval-process-cber/vaccine-development-101 cited 14 Feb 2021.

[35] Pollard AJ, Bijker EM (2021). A guide to vaccinology: from basic principles to new developments. Nat Rev Immunol.

[36] Centers for Disease Control and Prevention (CDC). Immunization: The Basics. Available from: https://www.cdc.gov/vaccines/vac-gen/imz-basics.htm

[37] Anderson RM, Vegvari C, Truscott J, Collyer BS (2020). Challenges in creating herd immunity to SARS-CoV-2 infection by mass vaccination. Lancet.

[38] Mallory ML, Lindesmith LC, Baric RS (2019). Vaccination-Induced Herd Immunity: Successes and challenges. J Allergy Clin Immunol.

[39] World Health Organization. How do vaccines work? Available from: https://www.who.int/news-room/feature-stories/detail/how-do-vaccines-work cited 20 Jan 2021.

[40] Sariol A, Perlman S (2020). Lessons for COVID-19 Immunity from Other Coronavirus Infections. Immunity.

[41] Frederiksen LSF, Zhang Y, Foged C, Thakur A (2020). The Long Road Toward COVID-19 Herd Immunity: Vaccine Platform Technologies and Mass Immunization Strategies. Front Immunol..

[42] Sridhar S, Brokstad K, Cox R (2015). Influenza Vaccination Strategies: Comparing Inactivated and Live Attenuated Influenza Vaccines. Vaccines.

[43] Lugade AA, Bharali DJ, Pradhan V, Elkin G, Mousa SA, Thanavala Y (2013). Single low-dose un-adjuvanted HBsAg nanoparticle vaccine elicits robust, durable immunity. Nanomedicine Nanotechnol Biol Med..

[44] van Riel D, de Wit E (2020). Next-generation vaccine platforms for COVID-19. Nat Mater..

[45] Cormier Z. The Second-Generation COVID Vaccines Are Coming. 2021; Available from: https://www.scientificamerican.com/article/the-second-generation-covid-vaccines-are-coming/

[46] Chapman R, Rybicki EP. Use of a Novel Enhanced DNA Vaccine Vector for Preclinical Virus Vaccine Investigation. Vaccines. 2019;7(2). Available from: https://www.mdpi.com/2076-393X/7/2/50 10.3390/vaccines7020050 PMC6632145 31200559

[47] Baruah V, Bose S (2020). Immunoinformatics-aided identification of T cell and B cell epitopes in the surface glycoprotein of 2019-nCoV. J Med Virol.

[48] Gupta T, Gupta SK (2020). Potential adjuvants for the development of a SARS-CoV-2 vaccine based on experimental results from similar coronaviruses. Int Immunopharmacol..

[49] Eidi H, Joubert O, Attik G, Duval RE, Bottin MC, Hamouia A (2010). Cytotoxicity assessment of heparin nanoparticles in NR8383 macrophages. Int J Pharm..

[50] Eidi H, Joubert O, Némos C, Grandemange S, Mograbi B, Foliguet B (2012). Drug delivery by polymeric nanoparticles induces autophagy in macrophages. Int J Pharm.

[51] Salvador A, Sandgren KJ, Liang F, Thompson EA, Koup RA, Pedraz JL (2015). Design and evaluation of surface and adjuvant modified PLGA microspheres for uptake by dendritic cells to improve vaccine responses. Int J Pharm.

[52] The National Institute for Public Health and the Environment. COVID-19 vaccination. Available from: https://www.rivm.nl/en/novel-coronavirus-covid-19/vaccine-against-covid-19 cited 2021 Oct 2021.

[53] Le Thanh T, Andreadakis Z, Kumar A, Gómez Román R, Tollefsen S, Saville M (2020). The COVID-19 vaccine development landscape. Nat Rev Drug Discov.

[54] Reuters. COVID-19 tracker. Latin America and the Caribbean. 2022. Available from: https://graphics.reuters.com/world-coronavirus-tracker-and-maps/regions/latin-america-and-the-caribbean/

[55] Reuteurs. COVID-19 Vaccination tracker. 2022. Available from: https://graphics.reuters.com/world-coronavirus-tracker-and-maps/vaccination-rollout-and-access/.

[56] Ritchie H, Mathieu E, Rodés-Guirao L, Appel C, Giattino C, Ortiz-Ospina E, et al. Coronavirus Pandemic (COVID-19). 2022. Available from: https://ourworldindata.org/covid-vaccinations?country=COL#citation

[57] Sharma O, Sultan AA, Ding H, Triggle CR (2019). A Review of the Progress and Challenges of Developing a Vaccine for COVID-19. Front Immunol.

[58] Roper RL, Rehm KE (2009). SARS vaccines: Where are we?. Expert Rev Vaccines.

[59] Enjuanes L, DeDiego ML, Álvarez E, Deming D, Sheahan T, Baric R (2008). Vaccines to prevent severe acute respiratory syndrome coronavirus-induced disease. Virus Res.

[60] Saif LJ, Wang Q, Vlasova AN, Jung K, Xiao S. Coronaviruses. In: Diseases of Swine. Wiley; 2019. p. 488–523. Available from: https://onlinelibrary.wiley.com/doi/abs/10.1002/9781119350927.ch31

[61] Schindewolf C, Menachery VD (2019). Middle east respiratory syndrome vaccine candidates: Cautious optimism. Viruses.

[62] Bolles M, Deming D, Long K, Agnihothram S, Whitmore A, Ferris M (2011). A Double-Inactivated Severe Acute Respiratory Syndrome Coronavirus Vaccine Provides Incomplete Protection in Mice and Induces Increased Eosinophilic Proinflammatory Pulmonary Response upon Challenge. J Virol.

[63] Tseng C-T, Sbrana E, Iwata-Yoshikawa N, Newman PC, Garron T, Atmar RL (2012). Immunization with SARS Coronavirus Vaccines Leads to Pulmonary Immunopathology on Challenge with the SARS Virus. PLoS One.

[64] Takasuka TE, Walker JA, Bergeman LF, Meulen KAV, Makino SI, Elsen NL (2014). Cell-free translation of biofuel enzymes. Methods Mol Biol.

[65] Lin J-T, Zhang J-S, Su N, Xu J-G, Wang N, Chen J-T (2007). Safety and immunogenicity from a phase I trial of inactivated severe acute respiratory syndrome coronavirus vaccine. Antivir Ther.

[66] Agrawal AS, Tao X, Algaissi A, Garron T, Narayanan K, Peng BH (2016). Immunization with inactivated the Middle East Respiratory Syndrome coronavirus vaccine leads to lung immunopathology on challenge with live virus. Hum Vaccines Immunother.

[67] Chen H, Guo J, Wang C, Luo F, Yu X, Zhang W (2020). Clinical characteristics and intrauterine vertical transmission potential of COVID-19 infection in nine pregnant women: a retrospective review of medical records. Lancet.

[68] Ciabattini A, Nardini C, Santoro F, Garagnani P, Franceschi C, Medaglini D (2018). Vaccination in the elderly: The challenge of immune changes with aging. Semin Immunol.

[69] Offit PA (2005). The Cutter Incident, 50 Years Later. N Engl J Med.

[70] Mulligan MJ (2020). An Inactivated Virus Candidate Vaccine to Prevent COVID-19. JAMA.

[71] Liang Z, Zhu H, Wang X, Jing B, Li Z, Xia X (2020). Adjuvants for Coronavirus Vaccines. Front Immunol..

[72] Vaccines. 2020. Available from: https://coronavirus.jhu.edu/vaccines cited 26 Nov 2020.

[73] Gao Q, Bao L, Mao H, Wang L, Xu K, Yang M (2020). Development of an inactivated vaccine candidate for SARS-CoV-2. Science (80-).

[74] Chua BY, Sekiya T, Jackson DC (2018). Opinion: Making Inactivated and Subunit-Based Vaccines Work. Viral Immunol.

[75] World Health Organization. The different types of COVID-19 vaccines. Available from: https://www.who.int/news-room/feature-stories/detail/the-race-for-a-covid-19-vaccine-explained cited 28 Feb 2021.

[76] Xia S, Duan K, Zhang Y, Zhao D, Zhang H, Xie Z, et al. Effect of an Inactivated Vaccine Against SARS-CoV-2 on Safety and Immunogenicity Outcomes. JAMA [Internet]. 2020 Sep 8;324(10):951. Available from: https://jamanetwork.com/journals/jama/fullarticle/2769612 10.1001/jama.2020.15543 PMC7426884 32789505

[77] Ada G (2005). Overview of vaccines and vaccination. Mol Biotechnol.

[78] Chen JW, Chen JM (2020). Potential of live pathogen vaccines for defeating the COVID-19 pandemic: History and mechanism. J Med Virol.

[79] Minor PD (2015). Live attenuated vaccines: Historical successes and current challenges. Virology.

[80] Ma Z, Li Z, Dong L, Yang T, Xiao S. Reverse genetic systems: Rational design of coronavirus live attenuated vaccines with immune sequelae. In: Advances in Virus Research. 2020. p. 383–416. Available from: https://linkinghub.elsevier.com/retrieve/pii/S006535272030021X 10.1016/bs.aivir.2020.06.003 PMC7326460 32711735

[81] Stern PL (2020). Key steps in vaccine development. Ann Allergy Asthma Immunol..

[82] Shin MD, Shukla S, Chung YH, Beiss V, Chan SK, Ortega-Rivera OA (2020). COVID-19 vaccine development and a potential nanomaterial path forward. Nat Nanotechnol.

[83] Gillim-Ross L, Subbarao K (2006). Emerging Respiratory Viruses: Challenges and Vaccine Strategies. Clin Microbiol Rev..

[84] Chen Y, Liu Q, Guo D (2020). Emerging coronaviruses: Genome structure, replication, and pathogenesis. J Med Virol.

[85] Nathanson N, Langmuir AD (1963). The cutter incident. poliomyelitis following formaldehyde-inactivated poliovirus vaccination in the united states during the spring of 1955. ii. relationship of poliomyelitis to cutter vaccine. Am J Hyg..

[86] Chen Y, Li L (2020). SARS-CoV-2: virus dynamics and host response. Lancet Infect Dis.

[87] Xing Y-H, Ni W, Wu Q, Li W-J, Li G-J, Wang W-D (2020). Prolonged viral shedding in feces of pediatric patients with coronavirus disease 2019. J Microbiol Immunol Infect.

[88] ThiNhu Thao T, Labroussaa F, Ebert N, V’kovski P, Stalder H, Portmann J (2020). Rapid reconstruction of SARS-CoV-2 using a synthetic genomics platform. Nature.

[89] Xie X, Muruato A, Lokugamage KG, Narayanan K, Zhang X, Zou J (2020). An Infectious cDNA Clone of SARS-CoV-2. Cell Host Microbe.

[90] Schoeman D, Fielding BC (2019). Coronavirus envelope protein: current knowledge. Virol J.

[91] DeDiego ML, Nieto-Torres JL, Jimenez-Guardeño JM, Regla-Nava JA, Castaño-Rodriguez C, Fernandez-Delgado R (2014). Coronavirus virulence genes with main focus on SARS-CoV envelope gene. Virus Res.

[92] Lamirande EW, DeDiego ML, Roberts A, Jackson JP, Alvarez E, Sheahan T (2008). A Live Attenuated Severe Acute Respiratory Syndrome Coronavirus Is Immunogenic and Efficacious in Golden Syrian Hamsters. J Virol.

[93] Menachery VD, Debbink K, Baric RS (2014). Coronavirus non-structural protein 16: Evasion, attenuation, and possible treatments. Virus Res.

[94] Robson F, Khan KS, Le TK, Paris C, Demirbag S, Barfuss P (2020). Coronavirus RNA Proofreading: Molecular Basis and Therapeutic Targeting. Mol Cell.

[95] Bull JJ (2015). Evolutionary reversion of live viral vaccines: Can genetic engineering subdue it?. Virus Evol.

[96] Shi P, Su Y, Li R, Liang Z, Dong S, Huang J (2019). PEDV nsp16 negatively regulates innate immunity to promote viral proliferation. Virus Res.

[97] Draft landscape of Covid-19 candidate vaccines. 2020. Available from: https://www.who.int/docs/default-source/a-future-for-children/novel-coronavirus_landscape_covid-19.pdf?sfvrsn=4d8bd201_1 cited 10 May 2021.

[98] Flanagan KL, Best E, Crawford NW, Giles M, Koirala A, Macartney K (2020). Progress and Pitfalls in the Quest for Effective SARS-CoV-2 (COVID-19) Vaccines. Front Immunol.

[99] Gavi. What are whole virus vaccines and how could they be used against COVID-19? 2020. Available from: https://www.gavi.org/vaccineswork/what-are-whole-virus-vaccines-and-how-could-they-be-used-against-covid-19 cited 24 Nov 2020.

[100] Mohsen MO, Zha L, Cabral-Miranda G, Bachmann MF (2017). Major findings and recent advances in virus-like particle (VLP)-based vaccines. Semin Immunol..

[101] Bezu L, Kepp O, Cerrato G, Pol J, Fucikova J, Spisek R (2018). Trial watch: Peptide-based vaccines in anticancer therapy. Oncoimmunol.

[102] Malonis RJ, Lai JR, Vergnolle O (2020). Peptide-Based Vaccines: Current Progress and Future Challenges. Chem Rev.

[103] Li W, Joshi M, Singhania S, Ramsey K, Murthy A (2014). Peptide Vaccine: Progress and Challenges. Vaccines.

[104] Amanat F, Krammer F (2020). SARS-CoV-2 Vaccines: Status Report. Immunity..

[105] Francis MJ (2018). Recent Advances in Vaccine Technologies. Vet Clin North Am Small Anim Pract.

[106] Liu DX, Fung TS, Chong KK-L, Shukla A, Hilgenfeld R (2014). Accessory proteins of SARS-CoV and other coronaviruses. Antiviral Res.

[107] Takashima Y, Osaki M, Ishimaru Y, Yamaguchi H, Harada A (2011). Artificial Molecular Clamp: A Novel Device for Synthetic Polymerases. Angew Chemie Int Ed.

[108] Zhang L, Wang W, Wang S (2015). Effect of vaccine administration modality on immunogenicity and efficacy. Expert Rev Vaccines..

[109] Medicago. COVID-19: Medicago’s Development Programs.. Available from: https://www.medicago.com/en/covid-19-programs/ cited 25 Aug 2020

[110] Zhang J, Zeng H, Gu J, Li H, Zheng L, Zou Q (2020). Progress and Prospects on Vaccine Development against SARS-CoV-2. Vaccines.

[111] Jiang T, Gao L, Lu J, Zhang Y-D (2013). ACE2-Ang-(1–7)-Mas Axis in Brain: A Potential Target for Prevention and Treatment of Ischemic Stroke. Curr Neuropharmacol.

[112] Ross K, Senapati S, Alley J, Darling R, Goodman J, Jefferson M (2019). Single dose combination nanovaccine provides protection against influenza A virus in young and aged mice. Biomater Sci.

[113] Bachmann MF, Jennings GT (2010). Vaccine delivery: a matter of size, geometry, kinetics, and molecular patterns. Nat Rev Immunol.

[114] Wang H, Zhang Y, Huang B, Deng W, Quan Y, Wang W (2020). Development of an Inactivated Vaccine Candidate, BBIBP-CorV, with Potent Protection against SARS-CoV-2. Cell.

[115] Baviskar T, Raut D, Bhatt LK (2021). Deciphering Vaccines for COVID-19: where do we stand today?. Immunopharmacol Immunotoxicol..

[116] Ura T, Okuda K, Shimada M (2014). Developments in Viral Vector-Based Vaccines. Vaccines.

[117] Du L, Zhao G, Lin Y, Sui H, Chan C, Ma S (2008). Intranasal Vaccination of Recombinant Adeno-Associated Virus Encoding Receptor-Binding Domain of Severe Acute Respiratory Syndrome Coronavirus (SARS-CoV) Spike Protein Induces Strong Mucosal Immune Responses and Provides Long-Term Protection against SARS-. J Immunol.

[118] Rauch S, Jasny E, Schmidt KE, Petsch B (2018). New vaccine technologies to combat outbreak situations. Front Immunol.

[119] A Study of Ad26.COV2.S for the Prevention of SARS-CoV-2-Mediated COVID-19 in Adult Participants (ENSEMBLE). Available from: https://clinicaltrials.gov/ct2/show/NCT04505722 cited 25 Aug 2020

[120] Milligan ID, Gibani MM, Sewell R, Clutterbuck EA, Campbell D, Plested E (2016). Safety and Immunogenicity of Novel Adenovirus Type 26– and Modified Vaccinia Ankara-Vectored Ebola Vaccines. JAMA.

[121] Different COVID-19 Vaccines. Available from: https://www.cdc.gov/coronavirus/2019-ncov/vaccines/different-vaccines.html cited 14 Feb 2020.

[122] Lu R, Zhao X, Li J, Niu P, Yang B, Wu H (2020). Genomic characterization and epidemiology of 2019 novel coronavirus: implications for virus origins and receptor binding. Lancet..

[123] Smith TRF, Patel A, Ramos S, Elwood D, Zhu X, Yan J (2020). Immunogenicity of a DNA vaccine candidate for COVID-19. Nat Commun.

[124] Kandeel M, Ibrahim A, Fayez M, Al-Nazawi M (2020). From SARS and MERS CoVs to SARS-CoV-2: Moving toward more biased codon usage in viral structural and nonstructural genes. J Med Virol.

[125] Weekly epidemiological update - 3 November 2020. Available from: https://www.who.int/publications/m/item/weekly-epidemiological-update---3-november-2020 cited 4 Nov 2020

[126] Fausther-Bovendo H, Kobinger GP (2014). Pre-existing immunity against Ad vectors: Humoral, cellular, and innate response, what’s important?. Hum Vaccines Immunother.

[127] Dicks MDJ, Spencer AJ, Edwards NJ, Wadell G, Bojang K, Gilbert SC (2012). A Novel Chimpanzee Adenovirus Vector with Low Human Seroprevalence: Improved Systems for Vector Derivation and Comparative Immunogenicity. PLoS One.

[128] Sanchez-Felipe L, Vercruysse T, Sharma S, Ma J, Lemmens V, Van Looveren D (2021). A single-dose live-attenuated YF17D-vectored SARS-CoV-2 vaccine candidate. Nature.

[129] Knuchel MC, Marty RR, Morin TNA, Ilter O, Zuniga A, Naim HY (2013). Relevance of a pre-existing measles immunity prior to immunization with a recombinant measles virus vector. Hum Vaccin Immunother.

[130] European Medicines Agency. COVID-19 Vaccine Janssen. 2021. Available from: https://www.ema.europa.eu/en/medicines/human/EPAR/covid-19-vaccine-janssen

[131] The Top 5 COVID-19 Vaccine Candidates Explained [Internet]. Available from: https://labblog.uofmhealth.org/rounds/top-5-covid-19-vaccine-candidates-explained cited 24 Oct 2020.

[132] Nayak S, Herzog RW (2010). Progress and prospects: immune responses to viral vectors. Gene Ther.

[133] Huang S, Kamihira M (2013). Development of hybrid viral vectors for gene therapy. Biotechnol Adv.

[134] Pandey A, Singh N, Vemula SV, Couëtil L, Katz JM, Donis R (2012). Impact of Preexisting Adenovirus Vector Immunity on Immunogenicity and Protection Conferred with an Adenovirus-Based H5N1 Influenza Vaccine. Subbiah E, editor. PLoS One..

[135] Yang Z, Wyatt LS, Kong W, Moodie Z, Moss B, Nabel GJ (2003). Overcoming Immunity to a Viral Vaccine by DNA Priming before Vector Boosting. J Virol..

[136] Martinon F, Krishnan S, Lenzen G, Magné R, Gomard E, Guillet J-G (1993). Induction of virus-specific cytotoxic T lymphocytesin Vivo liposome-entrapped mRNA. Eur J Immunol.

[137] Hobernik D, Bros M (2018). DNA Vaccines—How Far From Clinical Use?. Int J Mol Sci..

[138] Porter KR, Raviprakash K. DNA Vaccine Delivery and Improved Immunogenicity. Curr Issues Mol Biol. 2017;129–38. Available from: http://www.caister.com/cimb/abstracts/v22/129.html 10.21775/cimb.022.129 27831541

[139] de Queiroz NMGP, Marinho FV, Chagas MA, Leite LCC, Homan EJ, de Magalhães MTQ (2020). Vaccines for COVID-19: perspectives from nucleic acid vaccines to BCG as delivery vector system. Microbes Infect.

[140] Lazo L, Valdes I, Guillén G, Hermida L, Gil L (2019). Aiming at the heart: the capsid protein of dengue virus as a vaccine candidate. Expert Rev Vaccines.

[141] Pardi N, Hogan MJ, Porter FW, Weissman D (2018). mRNA vaccines — a new era in vaccinology. Nat Rev Drug Discov.

[142] Ulmer J, Donnelly J, Parker S, Rhodes G, Felgner P, Dwarki V (1993). Heterologous protection against influenza by injection of DNA encoding a viral protein. Science.

[143] Williams J (2013). Vector Design for Improved DNA Vaccine Efficacy, Safety, and Production. Vaccines..

[144] Liu M (2019). A Comparison of Plasmid DNA and mRNA as Vaccine Technologies. Vaccines..

[145] Callaway E. The race for coronavirus vaccines: a graphical guide. Vol. 580, Nature. England; 2020. p. 576–7. 10.1038/d41586-020-01221-y 32346146

[146] Alberer M, Gnad-Vogt U, Hong HS, Mehr KT, Backert L, Finak G (2017). Safety and immunogenicity of a mRNA rabies vaccine in healthy adults: an open-label, non-randomized, prospective, first-in-human phase 1 clinical trial. Lancet.

[147] Zhang C, Maruggi G, Shan H, Li J. Advances in mRNA Vaccines for Infectious Diseases. Front Immunol. 2019;10. 10.3389/fimmu.2019.00594/full 10.3389/fimmu.2019.00594 PMC6446947 30972078

[148] Lambricht L, Lopes A, Kos S, Sersa G, Préat V, Vandermeulen G (2016). Clinical potential of electroporation for gene therapy and DNA vaccine delivery. Expert Opin Drug Deliv.

[149] Liu L, Wei Q, Lin Q, Fang J, Wang H, Kwok H, et al. Anti–spike IgG causes severe acute lung injury by skewing macrophage responses during acute SARS-CoV infection. JCI Insight. 2019 Feb 21;4(4). Available from: https://insight.jci.org/articles/view/123158 10.1172/jci.insight.123158 PMC6478436 30830861

[150] Wang Z, Troilo PJ, Wang X, Griffiths TG, Pacchione SJ, Barnum AB (2004). Detection of integration of plasmid DNA into host genomic DNA following intramuscular injection and electroporation. Gene Ther.

[151] Schalk JAC, Mooi FR, Berbers GAM, van Aerts LAGJM, Ovelgönne H, Kimman TG (2006). Preclinical and Clinical Safety Studies on DNA Vaccines. Hum Vaccine.

[152] Tudor D, Dubuquoy C, Gaboriau V, Lefèvre F, Charley B, Riffault S (2005). TLR9 pathway is involved in adjuvant effects of plasmid DNA-based vaccines. Vaccine.

[153] Li L, Petrovsky N (2016). Molecular mechanisms for enhanced DNA vaccine immunogenicity. Expert Rev Vaccines.

[154] Yang Z, Kong W, Huang Y, Roberts A, Murphy BR, Subbarao K (2004). A DNA vaccine induces SARS coronavirus neutralization and protective immunity in mice. Nature.

[155] Qin C, Wang J, Wei Q, She M, Marasco WA, Jiang H (2005). An animal model of SARS produced by infection ofMacaca mulatta with SARS coronavirus. J Pathol.

[156] He Y, Li J, Li W, Lustigman S, Farzan M, Jiang S (2006). Cross-Neutralization of Human and Palm Civet Severe Acute Respiratory Syndrome Coronaviruses by Antibodies Targeting the Receptor-Binding Domain of Spike Protein. J Immunol.

[157] Cheng VCC, Lau SKP, Woo PCY, Kwok YY (2007). Severe acute respiratory syndrome coronavirus as an agent of emerging and reemerging infection. Clin Microbiol Rev.

[158] Kauffman KJ, Webber MJ, Anderson DG (2016). Materials for nonviral intracellular delivery of messenger RNA therapeutics. J Control Release.

[159] Coronavirus resource center. Types of COVID-19. Available from: https://coronavirus.jhu.edu/vaccines/reports/types-of-covid-19-vaccines cited 24 Nov 2020.

[160] Trafton A. Explained: Why RNA vaccines for Covid-19 raced to the front of the pack [Internet]. 2020. Available from: https://news.mit.edu/2020/rna-vaccines-explained-covid-19-1211 cited 10 May 2021.

[161] Cascella M, Rajnik M, Cuomo A, Dulebohn SC, Di Napoli R. Features, Evaluation, and Treatment Coronavirus (COVID-19). StatPearls. 2020; Available from: http://www.ncbi.nlm.nih.gov/pubmed/32150360 32150360

[162] Sahin U, Karikó K, Türeci Ö (2014). mRNA-based therapeutics — developing a new class of drugs. Nat Rev Drug Discov.

[163] Mulligan MJ, Lyke KE, Kitchin N, Absalon J, Gurtman A, Lockhart S (2020). Phase I/II study of COVID-19 RNA vaccine BNT162b1 in adults. Nature.

[164] Midoux P, Pichon C (2015). Lipid-based mRNA vaccine delivery systems. Expert Rev Vaccines.

[165] Zeng C, Hou X, Yan J, Zhang C, Li W, Zhao W, et al. Leveraging mRNAs sequences to express SARS-CoV-2 antigens in vivo. bioRxiv Prepr Serv Biol. 2020;32:2004452. 10.1002/adma.202004452 PMC8191860 32875709

[166] Moderna doses the first patient with mRNA-1273 in coronavirus vaccine trial. Available from: https://www.pharmaceutical-business-review.com/news/moderna-mrna-1273-coronavirus-trial/ cited 28 Feb 2021

[167] Safety and Immunogenicity Study of 2019-nCoV Vaccine (mRNA-1273) for Prophylaxis of SARS-CoV-2 Infection (COVID-19). Available from: https://clinicaltrials.gov/ct2/show/NCT04283461 cited 28 feb 2021.

[168] Zarghampoor F, Azarpira N, Khatami SR, Behzad-Behbahani A, Foroughmand AM (2019). Improved translation efficiency of therapeutic mRNA. Gene.

[169] Dolgin E. How COVID unlocked the power of RNA vaccines. 2021; Available from: https://www.nature.com/articles/d41586-021-00019-w 10.1038/d41586-021-00019-w 33437061

[170] Administration D. The Path for a COVID-19 Vaccine from Research to Emergency Use Authorization. 2021;19. Available from: www.FDA.gov/COVID19vaccines#FDAVaccineFacts

[171] Centers for Disease Control and Prevention (CDC). Interim Clinical Considerations for Use of COVID-19 Vaccines Currently Approved or Authorized in the United States. 2022. Available from: https://www.cdc.gov/vaccines/covid-19/clinical-considerations/covid-19-vaccines-us.html#booster-dose

[172] Jackson LA, Anderson EJ, Rouphael NG, Roberts PC, Makhene M, Coler RN (2020). An mRNA Vaccine against SARS-CoV-2 — Preliminary Report. N Engl J Med.

[173] Zhao L, Seth A, Wibowo N, Zhao C-X, Mitter N, Yu C (2014). Nanoparticle vaccines. Vaccine.

[174] Pati R, Shevtsov M, Sonawane A. Nanoparticle Vaccines Against Infectious Diseases. Front Immunol. 2018;9. 10.3389/fimmu.2018.02224/full 10.3389/fimmu.2018.02224 PMC6180194 30337923

[175] Vaccine BNT162b2 – Conditions of authorisation under Regulation 174 – 2 December 2020, amended on 30 December 2020, 28 January 2021, 30 March 2021, 19 May 2021, 04 June 2021, 29 July 2021, 9 September 2021, 27 September. 2021. Available from: https://www.gov.uk/government/publications/regulatory-approval-of-pfizer-biontech-vaccine-for-covid-19/conditions-of-authorisation-for-pfizerbiontech-covid-19-vaccine

[176] Wang J, Zand MS (2021). The potential for antibody-dependent enhancement of SARS-CoV-2 infection: Translational implications for vaccine development. J Clin Transl Sci.

[177] Greinacher A, Thiele T, Warkentin TE, Weisser K, Kyrle PA, Eichinger S. Thrombotic Thrombocytopenia after ChAdOx1 nCov-19 Vaccination. N Engl J Med. 2021;22:1–10. 10.1056/NEJMoa2104840 PMC8095372 33835769

